# Sijunzi decoction ameliorates geriatric sarcopenia by improving muscle strength, quality, and functions

**DOI:** 10.1097/MD.0000000000047154

**Published:** 2026-04-03

**Authors:** Yuebing Yue, Manfei Xu, Zheng Xu, Liyan Qian, Liping Dou, Jie Huang

**Affiliations:** a Department of Geratology, The Second Affiliated Hospital of Zhejiang Chinese Medical University, Gongshu District, Hangzhou, Zhejiang, China.

**Keywords:** inflammation, muscle strength, sarcopenia, Sijunzi decoction

## Abstract

**Background:**

To study the therapeutic efficacy and its mechanism of Sijunzi decoction (SJZD) in geriatric sarcopenia.

**Methods:**

Geriatric sarcopenia patients were divided into control group (n = 31) and research group (n = 31). Control group received basic intervention therapy, research group received basic intervention therapy and SJZD. Clinical efficacy (AMIS, 6 meters walking speed, and grip strength), comprehensive geriatric assessment (activities of daily living, Morse, mini-nutritional assessment, Frail, mini-mental state examination, self-rating anxiety scale, and geriatric depression scale), the functions of liver and kidney, blood cells inflammatory indexes (platelet-to-lymphocyte ratio, neutrophil-to-lymphocyte ratio, systemic immune-inflammatory index, systemic inflammation response index, and aggregate inflammation systemic index), and nutritional indicators were detected. Network pharmacology was used to study the mechanism of SJZD in treating sarcopenia.

**Results:**

After SJZD treatment, the levels of AMIS, 6 meters walking speed, and grip strength, the scores of activities of daily living, mini-nutritional assessment, and mini-mental state examination scale were significantly increased, compared with the control group, while the scores of Morse, Frail, self-rating anxiety scale, and geriatric depression scale were strongly decreased. The levels of aspartate aminotransferase, alanine aminotransferase, hemoglobin, albumin, prealbumin, and body mass index in research group were obviously higher than that in control group, but the levels of blood urea nitrogen, creatinine, platelet-to-lymphocyte ratio, neutrophil-to-lymphocyte ratio, systemic immune-inflammatory index, systemic inflammation response index, and aggregate inflammation systemic index were obviously decreased after treatment. Network pharmacology results showed that the active ingredients of SJZD acted on 36 targets of sarcopenia, which were mainly enriched in the interleukin-17 pathway, TNF pathway. We also found that the serum levels of interleukin-1β, matrix metalloprotein-9, tumor necrosis factor-α, interleukin-6, myostatin, and C-reactive protein were significantly reduced by SJZD, which suggested the roles of SJZD in inflammation.

**Conclusion:**

SJZD improved muscle strength, quality, and functions in sarcopenia patients by reducing peripheral blood inflammation.

## 1. Introduction

Sarcopenia is a geriatric syndrome characterized by age-related progressive, widespread loss of muscle mass and/or decreased muscle strength or muscle physiological functions.^[1,2]^ When the elderly suffer from sarcopenia, skeletal muscle mass, and strength are gradually lost, daily activities are restricted, easily leading to falls, fractures, and activity disorders. More importantly, sarcopenia in older adults causes a series of cardiovascular diseases or respiratory diseases, which seriously affects the life quality of older people, resulting in disability or death, even.^[3,4]^ Since the pathogenesis of sarcopenia has not been fully revealed, recent medicine only proposes treatment options based on the possible molecular mechanisms of sarcopenia and proves the feasibility of adjuvant therapies, including exercise therapy, nutritional therapy.^[5,6]^ However, there is no specific drug for the treatment of sarcopenia.^[7]^ Thus, it urgently needed to explore new treatment strategies for sarcopenia.

Traditional Chinese Medicine (TCM) has a long history in treating various diseases in clinic because of its safety and effectiveness. In TCM, sarcopenia can be defined as “impotence syndrome,”^[8,9]^ and treated based on the theory of “Spleen-invigorating and Qi-replenishing.”^[10,11]^ Sijunzi decoction (SJZD) is a classic formula in TCM, containing *Panax ginseng* C. A. Mey. (Renshen in Chinese), *Poria cocos (Schw.) Wolf* (Fuling in Chinese), *Atractylodes macrocephala Koidz*. (Baizhu in Chinese), and *Glycyrrhizae Radix Et Rhizoma Praeparata Cum Melle* (Gancao in Chinese). This formula can be used to improve the strength of spleen and the energy of whole body.^[12,13]^ Newest research mentioned that SJZD can ameliorate some aging diseases, such as premature ovarian insufficiency,^[14]^ skin aging.^[15]^ Nevertheless, the potential mechanism of SJZD in preventing geriatric sarcopenia has not been elucidated.

In this study, we observed the clinical efficacy of SJZD in patients with sarcopenia to assess whether SJZD treatment could enhance the efficacy of exercise therapy and nutritional therapy. At the same time, we used network pharmacology to explore the molecular mechanisms of SJZD in the treatment of sarcopenia.

## 2. Patients and methods

### 2.1. Participants and ethics statement

A total of 62 patients with geriatric sarcopenia were admitted to the Second Affiliated Hospital of Zhejiang Chinese Medical University from January 1, 2021 to December 31, 2022 with the completed clinical data and initial surgical treatment (grant no. 2022-KL-092-09). All patients with sarcopenia must meet the following criteria: first, the appendicular skeletal muscle mass index (ASMI) of male <7.0 kg/m^2^, and the ASMI of female <5.4 kg/m^2^. Second, 6 meters walking speed (6MWS) <0.8 m/s. Third, the grip strength of male <26 kg, and the grip strength of female <18 kg. And the study was approved by the Ethics Committee of the Second Affiliated Hospital of Zhejiang Chinese Medical University.

Inclusion criteria: 60 years < age < 90 years. The clinical data were complete and the 1-year follow-up was completed. Mental clarity and normal intelligence, and has good communication and cognitive abilities. Signed informed consent form to voluntarily join this study.

Exclusion criteria: History of dysfunction of other severe organs, impaired consciousness, and psychiatric disorders. Infectious diseases. Long term bed rest, intellectual disability, or mental illness that prevents normal communication. Patients receiving tumor radiotherapy, chemotherapy, or end-stage disease treatment. Participate in clinical trials of other drugs midway through the study.

### 2.2. Interventions

Patients in control group received 12 weeks of basic intervention therapy, and patients in research group received 12 weeks of basic intervention therapy and 12 weeks of SJZD treatment.

Basic intervention therapy included nutritional support and physical activity. The nutritional support program is formulated by the professional dietitian of the nutrition department of the hospital, which ensures that the patients with sarcopenia take appropriate and sufficient energy, protein and vitamins by evaluating the patient’s body, weight and other indicators, calculating the calories and nutrients required by the body, and through reasonable dietary matching. Physical exercise is selected by the rehabilitation therapist according to the patient’s physical state, and improves muscle strength and function through walking, doing health exercises or professional rehabilitation training.

SJZD, including ginseng, atractylodes, wolfiporia, and glycyrrhiza uralensis,^[16,17]^ were extracted with distilled water and ground into powders by a vacuum freeze dryer. Then, the SJZD powder was made into granules (1.77 g/pack) by mixing the powders in the adequate dosage ratio and adding dextrin. Patients were asked to take one package of SJZD orally by dissolving in 150 mL of hot water, 2 times a day before meals (3.54 g/d), for a total of 3 months. SJZD granules were provided by Sichuan Neo-green Pharmaceutical Technology Development Co., Ltd. (Sichuan, China).

### 2.3. Primary outcomes

After completing 12 weeks of treatment, we evaluated clinical efficacy by measuring muscle mass (ASMI), muscle strength (grip strength) and function (6MWS), and comprehensive geriatric assessment (CGA) in patients with sarcopenia. CGA included^[18]^: assessment of physical function status using the activities of daily living (ADL) scale. Evaluate nutritional status using the mini-nutritional assessment (MNA) scale. Frail scale assesses frailty. Mini-mental state examination (MMSE) scale, self-rating anxiety scale (SAS), and geriatric depression scale (GDS) are used to assess mental state.

### 2.4. Secondary outcomes

Secondary outcomes included 4 aspects: serum aspartate aminotransferase and alanine aminotransferase were used to evaluate liver function, while blood urea nitrogen (BUN) and creatinine were used to evaluate renal function. Blood cell inflammation index including platelet-to-lymphocyte ratio, neutrophil-to-lymphocyte ratio, systemic immune-inflammatory index, systemic inflammation response index and aggregate inflammation systemic index (AISI).^[19]^ Detecting hemoglobin, albumin, prealbumin, and body mass index to assess nutritional status. Serum levels of key proteins in interleukin-17 (IL-17) and TNF pathway, such as interleukin-1β (IL-1β), matrix metalloprotein-9 (MMP-9), tumor necrosis factor-α (TNF-α), interleukin-6 (IL-6), myostatin (MSTN), and C-reactive protein (CRP).

### 2.5. Identification of the targets of SJZD in sarcopenia

First of all, we screened in TCMSP database (https://lsp.nwu.edu.cn) to find out the active ingredients of SJZD, and the filtering criteria are specified as oral bioavailability ≥ 30% and drug likeness ≥ 0.18. At the same time, we searched the protein targets for the active ingredients of SJZD in TCMSP database.^[20]^ Next, in the Genecards (https://www.genecards.org), OMIM (https://omim.org), TTD (https://db.idrblab.net/ttd), and PharmaGKB (https://www.pharmgkb.org) databases, we searched sarcopenia-related genes/targets using the keyword of sarcopenia, and deleted duplicate genes/targets. At last, excel was used to get a “SJZD – sarcopenia” intersectional target database, and visualized using a Venn diagram (https://bioinfogp.cnb.csic.es/tools/venny/index.html).

### 2.6. Function and pathway enrichment

The intersection targets of SJZD and sarcopenia were imported into STRING database (https://string-db.org) to construct a protein-protein interaction (PPI) network model using biological species set to “Homo sapiens,” the confidence >0.4 and the rest of the parameters are set to default values. Then the top 10 hub genes were screened using the cytoHubba plug-in in Cytoscape software (U.S. National Human Genome Research Institute) base on the result of PPI network. Gene ontology and kyoto encyclopedia of genes and genomes pathway functional enrichment was analyzed on the STRING platform.

### 2.7. Statistical analysis

Data in the present study were recorded and analyzed in SPSS 20.0 (IBM, Chicago). Continuous data tables conforming to the normal distribution were presented as (mean ± standard deviation), and the differences between the 2 groups were compared by the Student *t* test. Continuous data tables that did not conform to the normal distribution were presented as median (interquartile range) and the differences between the 2 groups were compared by the Mann–Whitney test. Categorical variables were presented as percentages, and differences between the 2 groups were compared by Chi-square test. *P* < .05 indicates significant difference.

## 3. Results

### 3.1. Patient enrollment

**Figure 1.**
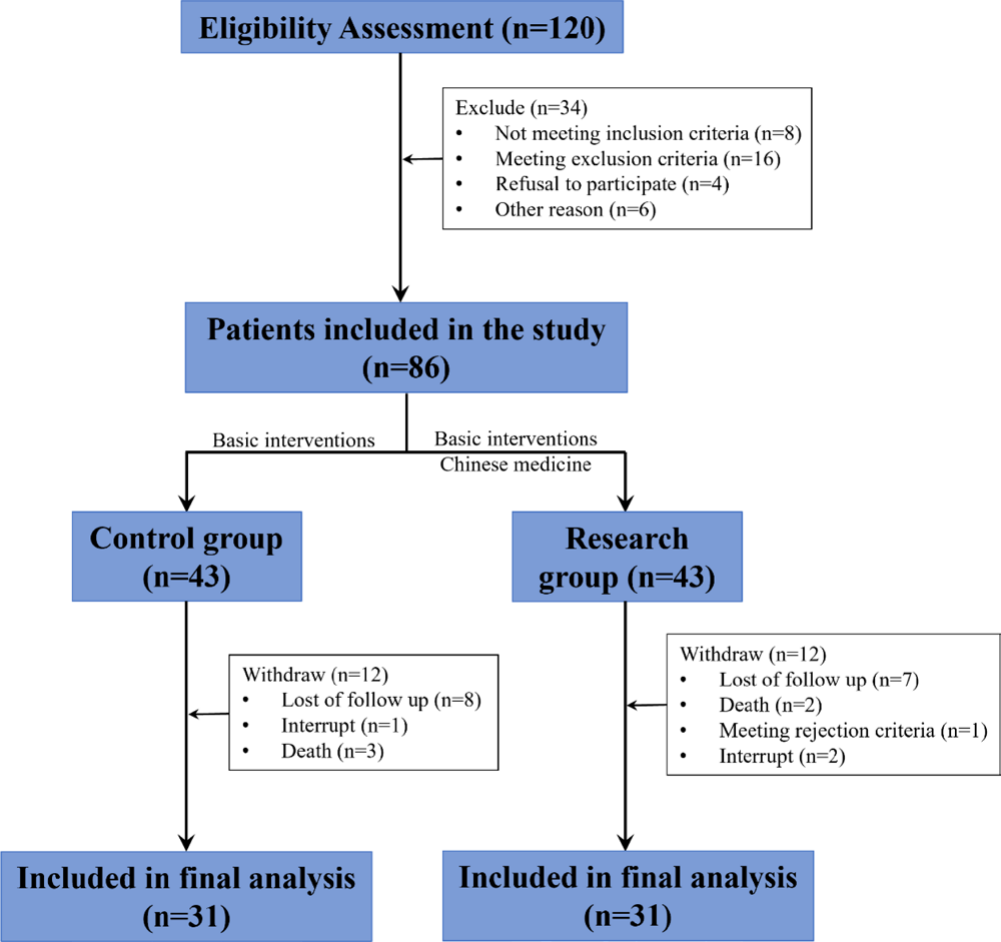
Flow chart for patient recruitment and analysis.

A total of 62 patients with geriatric sarcopenia were recruited and enrolled in the finally analysis, divided into 2 groups: control group (n = 31) and research group (n = 31) (Fig. 1). After treatment, we found that there was no significant difference in age, weight, body mass index, gender, waist-to-hip ratio, systolic blood pressure, diastolic blood pressure, smoking, drinking, hypertension, diabetes, cardiovascular disease, spouse death, and educational background between patients in control group research group (Table 1).

**Table 1:** Baseline characteristics of 62 elderly patients with sarcopenia.

	Control group (n = 31)	Research group (n = 31)	*t*/χ^2^	*P*-value
Age (yr)	71.52 ± 4.84	72.45 ± 4.95	0.753	.454
Weight (kg)	53.35 ± 8.33	56.32 ± 8.82	1.362	.178
BMI (kg/m^2^)	19.62 ± 0.96	19.33 ± 0.72	1.713	.087
Male (n [%])	16 (51.61)	14 (45.16)	0.258	.611
WHR	0.81 ± 0.76	0.82 ± 0.78	0.062	.967
SBP (mm Hg)	125.30 ± 16.42	128.74 ± 13.25	0.813	.468
DBP (mm Hg)	81.03 ± 10.89	82.16 ± 11.67	0.913	.431
Smoking (n [%])	12 (38.71)	14 (45.16)	0.265	.607
Drinking (n [%])	13 (41.94)	12 (38.71)	0.067	.796
Hypertension (n [%])	9 (29.03)	11 (35.48)	0.295	.587
Diabetes (n [%])	5 (16.13)	7 (22.58)	0.413	.520
Cardiovascular disease (n [%])	19 (61.29)	17 (54.84)	0.265	.407
Spouse death (n [%])	6 (19.35)	8 (25.81)	0.369	.544
High school and above (n [%])	5 (16.13)	6 (19.35)	0.111	.740

BMI = body mass index, DBP = diastolic blood pressure, SBP = systolic blood pressure, WHR = waist-to-hip ratio.

### 3.2. Primary outcomes: clinical efficacy

Before treatment, there was no significant difference between control group and research group on the levels of ASMI, 6MWS, and grip strength (*P* > .05). After treatment, the levels of ASMI, 6MWS, and grip strength in the 2 groups were significantly increased (*P* < .05). However, this increase of ASMI, 6MWS, and grip strength in research group were significantly higher than that of the control group (*P* < .05) (Table 2). In addition, there was also no significant difference between these 2 group on the score of ADL, Morse, MNA, Frail, MMSE, SAS, and GDS (*P* > .05). After SJZD treatment, all signals were significantly changed (*P* < .05), the score of ADL, MNA, and MMSE scale in patients of research group were strongly higher than that in patients of control group (*P* < .05), while the score of Morse, Frail, SAS, and GDS in patients of research group were remarkably lower than that in patients of control group after treatment (*P* < .05) (Table 3).

**Table 2:** Comparison of muscle related indicators between 2 groups (mean ± SD).

	Control group		Research group	
	Before treatment	After treatment	Before treatment	After treatment
AMSI (kg/m^2^)	5.05 ± 0.32	5.18 ± 0.43†	5.07 ± 0.28^ns^	5.63 ± 0.42*^,^†
6MWS (m/s)	0.50 ± 0.03	0.65 ± 0.03†	0.52 ± 0.02^ns^	0.75 ± 0.03*^,^†
Grip strength (kg)	16.46 ± 2.91	19.25 ± 2.53†	17.46 ± 2.88^ns^	21.98 ± 2.58*^,^†

6MWS = 6 meters walking speed, ASMI = appendicular skeletal muscle mass index.

ns Compared with control group, *P* > .05.

* Compared with control group, *P* < .05.

† Compared with before treatment in the same group, *P* < .05.

**Table 3:** Comparison of comprehensive geriatric assessment between 2 groups (mean ± SD).

	Control group		Research group	
	Before treatment	After treatment	Before treatment	After treatment
ADL	68.92 ± 5.33	71.35 ± 5.94†	68.79 ± 7.65^ns^	74.25 ± 6.58*^,^†
Morse	35.21 ± 3.06	32.38 ± 4.32†	35.29 ± 5.65^ns^	30.56 ± 3.05*^,^†
MNA	5.25 ± 1.54	6.09 ± 2.04†	5.39 ± 1.95^ns^	6.98 ± 2.12*^,^†
Frail	2.33 ± 0.94	2.04 ± 0.62†	2.37 ± 0.74^ns^	1.81 ± 0.63*^,^†
MMSE	22.65 ± 4.41	23.38 ± 5.17†	22.74 ± 5.62^ns^	24.91 ± 3.12*^,^†
SAS	38.62 ± 3.52	36.42 ± 4.11†	39.05 ± 4.25^ns^	34.27 ± 3.64*^,^†
GDS	8.05 ± 1.38	7.11 ± 2.42†	8.10 ± 2.15^ns^	6.02 ± 1.15*^,^†

ADL = activities of daily living, GDS = geriatric depression scale, MMSE = mini-mental state examination, MNA = mini-nutritional assessment, SAS = self-rating anxiety scale.

ns Compared with control group, *P* > .05.

* Compared with control group, *P* < .05.

† Compared with before treatment in the same group, *P* < .05.

### 3.3. Secondary outcomes

The functions of liver and kidney were used to assess drug toxicity. In this work, we found that there was no significant difference in the levels of liver and kidney functional markers between the control group and the research group before treatment. The basic intervention therapy (nutrition and physical activity) only obviously reduced the level of BUN in control group. However, SJZD treatment not only strongly increased the level of aspartate aminotransferase and alanine aminotransferase in the research group, but also noticeably reduced the level of BUN and creatinine in the research group (Table 4). At the same time, we also studied the effects of different treatments on blood cells inflammatory index and the status of nutrition. The observation indicated that both treatment protocols could reduce the inflammatory index of blood cells (*P* < .05), and inflammatory index in the research group were significantly lower than those in the control group (*P* < .05), including the levels of platelet-to-lymphocyte ratio, neutrophil-to-lymphocyte ratio, systemic immune-inflammatory index, systemic inflammation response index, and AISI (Table 5). Similarly, both treatment protocols could obviously increase nutrition-related indicators, and the improvement in SJZD treatment group were more notable than those in the control group (*P* < .05), including levels of hemoglobin, albumin, prealbumin, and AISI (Table 6).

**Table 4:** Comparison of AST, ALT, BUN, and Cr between 2 groups (mean ± SD).

	Control group		Research group	
	Before treatment	After treatment	Before treatment	After treatment
AST (U/L)	12.39 ± 4.44	11.55 ± 3.60†	11.81 ± 5.86ns	13.87 ± 4.96†^,^*
ALT (U/L)	12.13 ± 4.50	10.26 ± 3.44†	11.52 ± 6.54ns	14.61 ± 3.95‡^,^*
BUN (mmol/L)	6.47 ± 1.12	5.05 ± 0.99‡	6.98 ± 0.84ns	4.85 ± 0.70‡^,^ns
Cr (μmol/L)	73.32 ± 5.04	75.58 ± 4.97†	74.08 ± 6.19ns	62.71 ± 7.87‡^,^*

ALT = alanine aminotransferase, AST = aspartate aminotransferase, BUN = blood urea nitrogen, Cr = creatinine.

nsCompared with control group, *P* > .05.

* Compared with control group, *P* < .05.

† Compared with before treatment in the same group, *P* < .05.

‡ Compared with before treatment in the same group, *P* < .01.

**Table 5:** Comparison of blood cells inflammatory index between 2 groups (mean ± SD).

	Control group		Research group	
	Before treatment	After treatment	Before treatment	After treatment
PLR	142.35 ± 35.21	135.62 ± 38.12†	144.18 ± 40.63ns	126.25 ± 37.54†^,^*
NLR	2.62 ± 0.65	2.24 ± 0.47†	2.65 ± 0.73^ns^	1.96 ± 0.59†^,^*
SII	425.14 ± 89.71	400.57 ± 80.25†	426.81 ± 93.54^ns^	384.35 ± 86.79†^,^*
SIRI	1.16 ± 0.43	1.05 ± 0.38†	1.17 ± 0.51^ns^	0.92 ± 0.46†^,^*
AISI	193.65 ± 57.21	183.35 ± 36.79†	194.67 ± 60.02^ns^	169.68 ± 45.81†^,^*

AISI = aggregate inflammation systemic index, NLR = neutrophil-to-lymphocyte ratio, PLR = platelet-to-lymphocyte ratio, SII = systemic immune-inflammatory index, SIRI = systemic inflammation response index.

nsCompared with control group, *P* > .05.

* Compared with control group, *P* < .05.

† Compared with before treatment in the same group, *P* < .01.

**Table 6:** Comparison of nutrition-related indicators between 2 groups (mean ± SD).

	Control group		Research group	
	Before treatment	After treatment	Before treatment	After treatment
Hb (g/L)	112.83 ± 12.03	120.09 ± 11.56†	111.97 ± 1116ns	128.97 ± 14.65†^,^*
Alb (g/L)	30.35 ± 4.65	31.89 ± 5.21†	30.19 ± 4.72ns	33.86 ± 5.16†^,^*
Plb (mg/L)	170.65 ± 52.36	175.12 ± 35.68†	169.84 ± 46.38ns	182.32 ± 54.02†^,^*
BMI (kg/m^2^)	19.62 ± 0.96	21.47 ± 1.89†	19.33 ± 0.72ns	20.19 ± 1.48†^,^*

Alb = albumin, BMI = body mass index, Hb = hemoglobin, Plb = prealbumin.

nsCompared with control group, *P* > .05.

* Compared with control group, *P* < .05.

† Compared with before treatment in the same group, *P* < .01.

### 3.4. Targets of SJZD in sarcopenia

**Figure 2.**
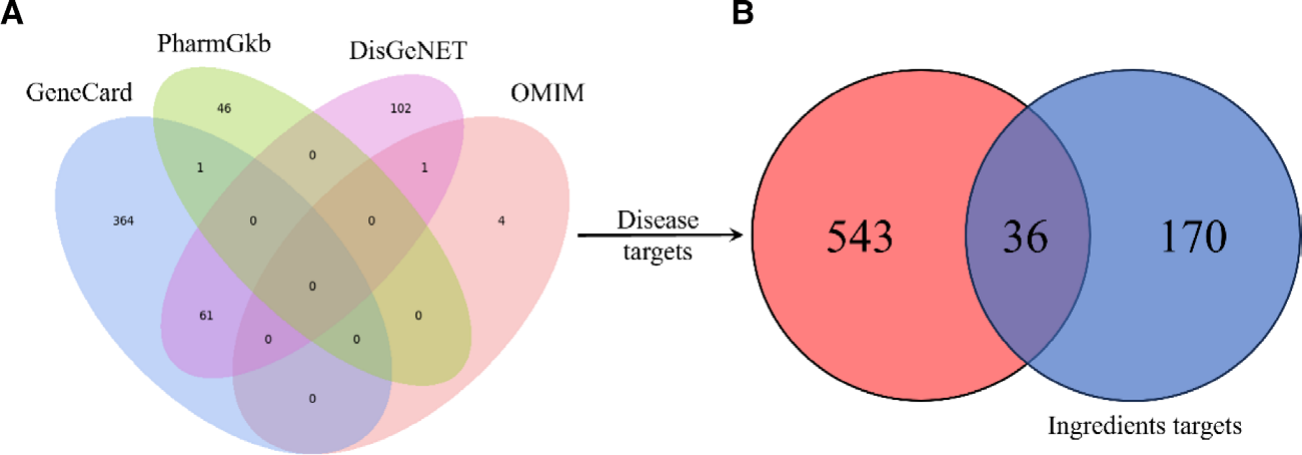
Targets of Sijunzi decoction and sarcopenia. (A) Venn diagram of the Sijunzi decoction according to Genecard, PhamGkb, DisGeNET, and OMIM databases. (B) The targets of sarcopenia and active ingredients targets of Sijunzi decoction.

**Figure 3.**
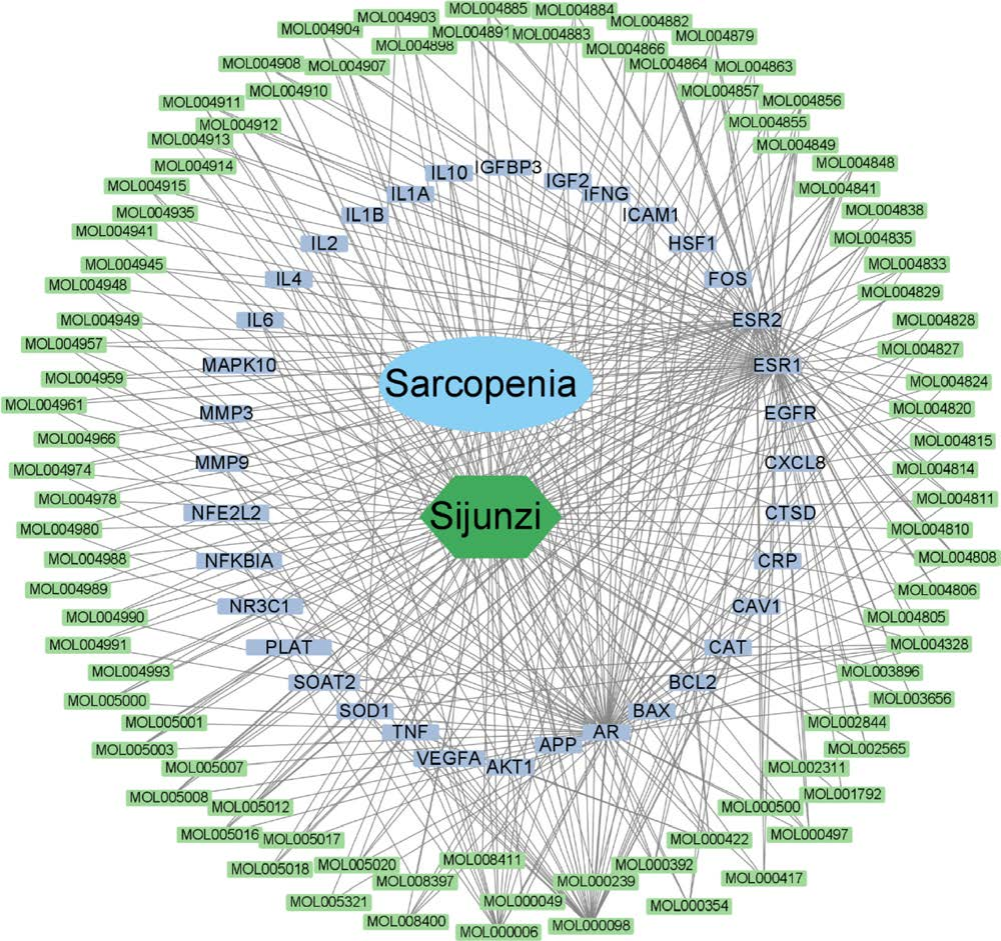
Active ingredients of Sijunzi decoction act on the target of sarcopenia.

In GeneCard, PharmGkb, DisGeNET, and OMIM databases, we have identified 426, 47, 165, and 5 targets for sarcopenia, respectively, and we confirmed 579 sarcopenia targets after removing duplicate ones (Fig. 2A). In addition, we identified 206 targets of 136 active ingredients in SJZD, and 36 intersected targets were presented in the Venn diagram between the targets of sarcopenia and SJZD (Fig. 2B). Then an interaction network diagram of “Drug-Target-Disease” based on 136 active ingredients of SJZD, sarcopenia, and 136 targets of sarcopenia (Fig. 3) was conducted to overview the functional factors of SJZD in sarcopenia.

### 3.5. Functional enrichment of 36 targets of SJZD

**Figure 4.**
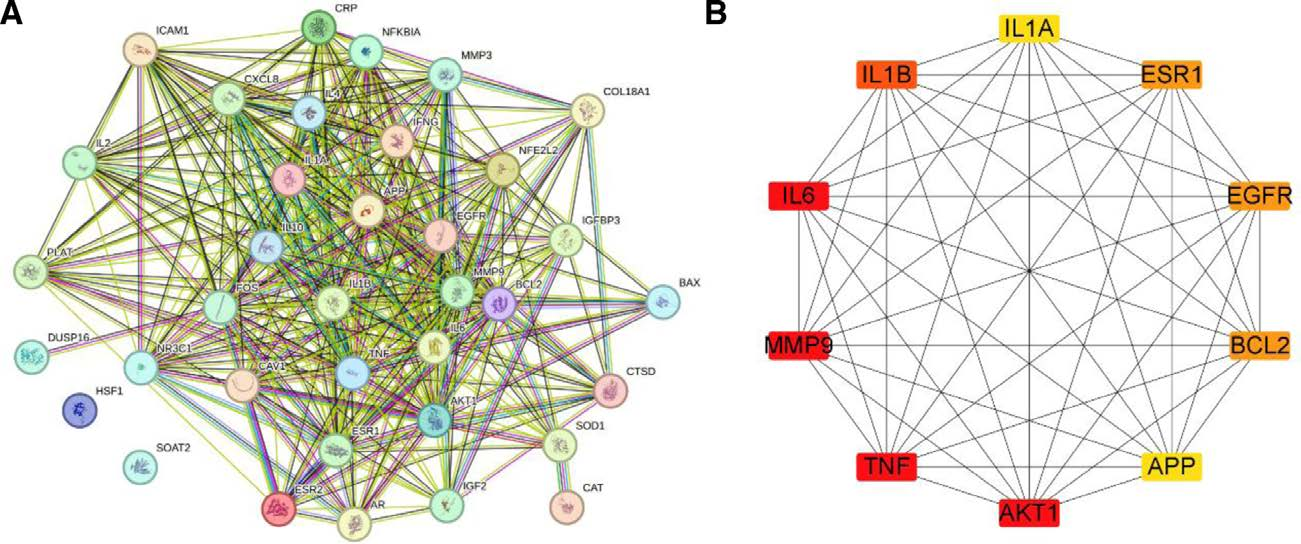
The interaction of hub gene in sarcopenia. (A) PPI network of 36 sarcopenia targets. (B) Top 10 hub targets. PPI = protein-protein interaction.

**Figure 5.**
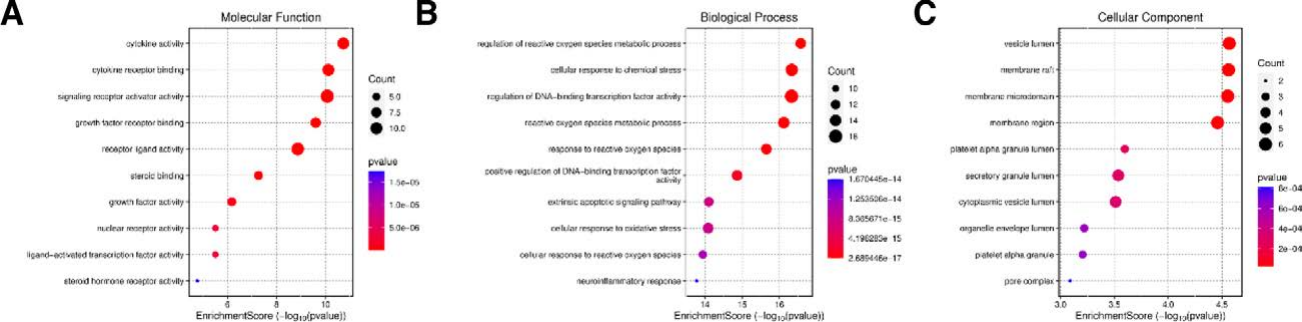
Function enrichment of 36 sarcopenia targets in GO database. (A) Molecular function. (B) Biological process. (C) Cellular component. GO = gene ontology.

**Figure 6.**
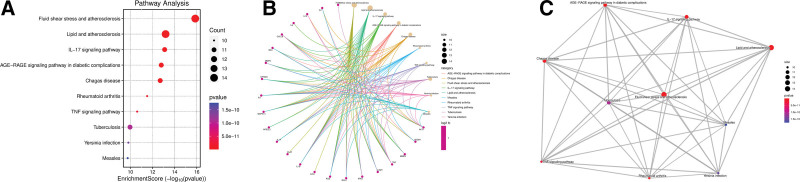
Pathway enrichment of KEGG database. (A) The main regulatory pathways were presented as dotplot. (B) The cnetplot. (C) The emapplot. KEGG = kyoto encyclopedia of genes and genomes.

**Figure 7.**
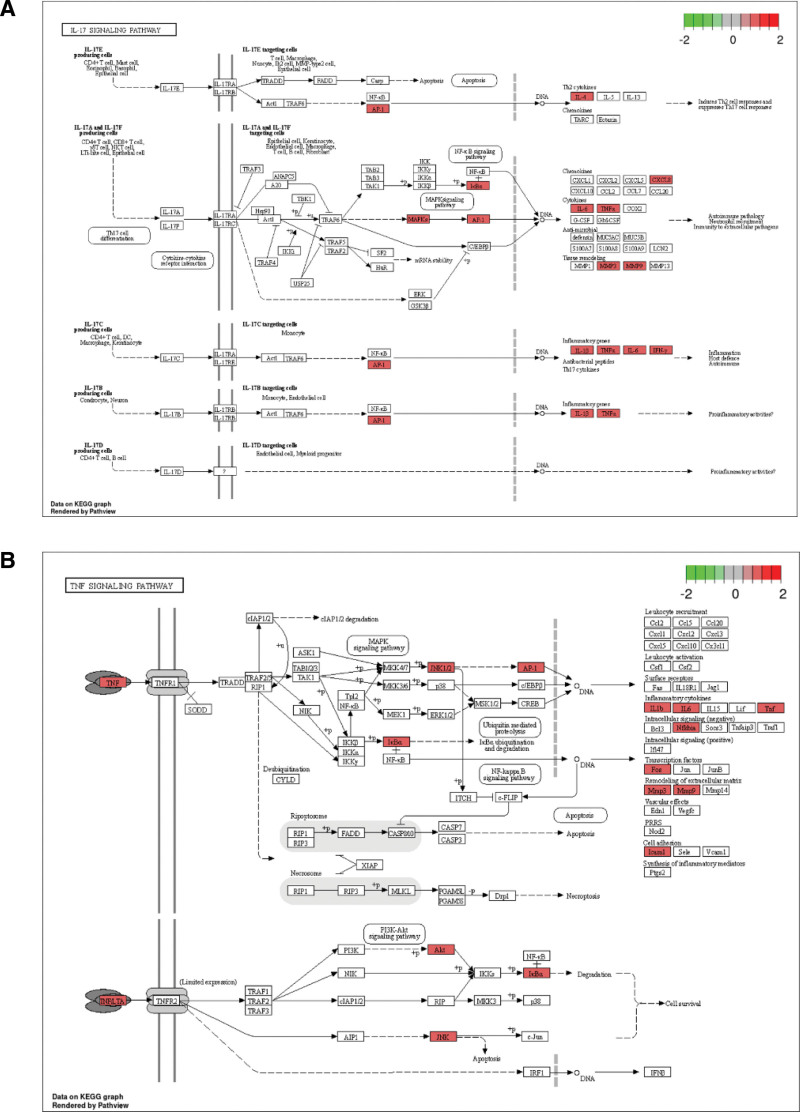
KEGG signaling pathways. (A) The IL-17 signaling pathway from KEGG database was presented and the potential targets among 36 sarcopenia targets were labeled in red. (B) The TNF signaling pathway from KEGG database was presented. The potential targets among 36 sarcopenia targets were labeled in red. IL-17 = interleukin-17, KEGG = kyoto encyclopedia of genes and genomes.

We used 36 sarcopenia targets, which were also the targets of SJZD, to build a PPI network using STRING database (Fig. 4A). The top 10 hub genes were screened using the cytoHubba plug-in in Cytoscape software base on the result of PPI network, namely, IL-6, MMP-9, TNF, protein kinase B alpha, IL1B (IL-1β), B-cell lymphoma 2, epidermal growth factor receptor, estrogen receptor alpha gene, IL1A (IL-1α), and APP (Fig. 4B). The enrichment analysis in gene ontology database showed that the key molecular function, biological process and cellular component pathways for SJZD intervention sarcopenia, including cytokine activity cytokine receptor binding, regulation of reactive oxygen species metabolic process, cellular response to chemical stress, vesicle lumen, and membrane raft (Fig. 5). Kyoto encyclopedia of genes and genomes enrichment showed that fluid shear stress and atherosclerosis, IL-17 signaling pathway, advanced glycation end products and their receptor‌ signaling pathway in diabetic complications, TNF signaling pathway, and so on are the main pathways that SJZD affected sarcopenia (Fig. 6). Among these pathways, we supposed that the inflammatory-related pathways, like IL-17 signaling pathway (Fig. 7A) and TNF signaling pathway (Fig. 7B) may be important in the treatment of sarcopenia with SJZD.

### 3.6. Effect of SJZD through IL-17 and TNF pathways in sarcopenia

To confirmed whether SJZD improved sarcopenia through IL-17 signaling pathway and TNF signaling pathway, we detected the serum levels of several key proteins in the IL-17 and TNF signaling pathways, such as IL-1β, MMP-9, TNF-α, IL-6, MSTN, and CRP. We found that the serum levels of IL-1β, MMP-9, TNF-α, IL-6, MSTN, and CRP in control group were all decreased compared to before treatment. More importantly, the research treatment more significantly reduced the serum levels of IL-1β, MMP-9, TNF-α, IL-6, MSTN, and CRP, compared to before treatment and control ones (*P* < .05) (Table 7).

**Table 7:** Comparison of serum inflammatory cytokines between 2 groups (mean ± SD).

	Control group		Research group	
	Before treatment	After treatment	Before treatment	After treatment
IL-1β (ng/mL)	4.35 ± 1.12	3.02 ± 0.89‡	4.41 ± 1.09ns	2.58 ± 0.57‡^,^*
MMP-9 (ng/mL)	223.25 ± 69.78	200.97 ± 57.12‡	226.71 ± 72.56ns	182.39 ± 54.71‡^,^*
TNF-α (pg/mL)	20.56 ± 6.72	19.64 ± 5.43†	23.76 ± 7.60ns	16.97 ± 6.27‡^,^*
IL-6 (pg/mL)	55.18 ± 11.63	40.56 ± 8.08‡	58.41 ± 8.61ns	35.85 ± 5.49‡^,^*
MSTN (ng/mL)	24.86 ± 10.50	14.20 ± 6.77‡	23.83 ± 8.95ns	11.70 ± 4.27‡^,^*
CRP (mg/L)	8.07 ± 1.60	4.83 ± 1.61‡	8.12 ± 1.31ns	3.58 ± 1.14‡^,^*

CRP = C-reactive protein. IL-1β = interleukin-1β, IL-6 = interleukin-6, MMP-9 = matrix metalloprotein-9, MSTN = myostatin, TNF-α = tumor necrosis factor-α.

ns Compared with control group, *P* > .05.

* Compared with control group, *P* < .05.

† Compared with before treatment in the same group, *P* < .05.

‡ Compared with before treatment in the same group, *P* < .01.

## 4. Discussion

Sarcopenia is an age-related disorder with a high incidence in older adults, and its clinical manifestations are mainly decreased muscle mass, muscle strength, and muscle function.^[1,2]^ The Asian Working Group on Sarcopenia conducted an epidemiological study on sarcopenia in 2019, and found that the current prevalence rate ranges from 5.5% to 25.7%.^[5]^ The incidence of sarcopenia is increasing year by year with the intensification of aging, and the number of patients with sarcopenia is expected to reach 500 million by 2050.^[21,22]^ The lack of specific drugs is a difficult point in the treatment of sarcopenia, because only nutritional and exercise therapies are used to intervene in patients with sarcopenia. In classical TCM theory, the clinical manifestations of sarcopenia patients are defined as “impotence syndrome”^[8,9]^ and treated according to the theory of “Spleen-invigorating and Qi-replenishing.”^[10,11]^

In this study, we found that SJZD significantly improved the clinical efficacy of nutritional therapy and exercise therapy in patients with sarcopenia, that is, increased the level of ASMI, 6MWS, and grip strength, as well as the score of CGA. Although there are no studies reporting the therapeutic efficacy of SJZD in the treatment of sarcopenia patients, the results of relevant animal studies have shown that SJZD can improve the symptoms by regulating the expression of SIRT1, IL-6, TNF, and protein kinase B alpha in a rat model of lipopolysaccharide-induced sarcopenia.^[23]^ At the same time, SJZD had been found to improve the pathological changes of muscle mitochondria and neuromuscular junctions in autoimmune myasthenia gravis rats, thereby enhancing the ATP content of gastrocnemius muscle mitochondria and ultimately improving muscle quality and function.^[24]^ Taken together, SJZD can be used to treat patients with sarcopenia through improving muscle strength, quality, and function.

In terms of mechanism, the diversity of TCM ingredients is the material basis for its extensive pharmacological effects, but this makes it difficult to elucidate the specific molecular mechanisms of TCM in treating sarcopenia.^[25]^ Therefore, network pharmacology provides new ideas for TCM research. Network pharmacology is a new discipline based on the theory of systems biology, which analyzes the network of biological systems and selects specific signal nodes for multitarget drug molecular design.^[26]^ It has been used to assess the relationship between TCM and diseases in different fields such as discovering new drugs,^[27]^ elucidate pharmacological mechanisms,^[28]^ and explore new targets.^[29]^ Herein, we found that SJZD acted on 36 targets for sarcopenia, and these 36 targets were mainly enriched in IL-17 pathway and TNF pathway. Results of network pharmacology suggested that inflammation might be the key to the treatment of sarcopenia with SJZD. Combining previous research reports, Li et al found that SJZD could significantly reduce the expression levels of inflammatory cytokines such as IL-1β, IL-6, and TNF-α in mice with ulcerative colitis, and improve their nutritional status.^[30]^ Zhang et al found that the active ingredients ginsenosides Rh2, isoflavones, which were extracted from SJZD, could increase the body weight of ulcerative colitis mice by reducing neutrophil-associated inflammation.^[31]^ We further provided the experimental confirmation that SJZD treatment significantly reduced peripheral blood inflammation index through reduced the serum levels of IL-1β, MMP-9, TNF-α, IL-6, MSTN, and CRP and improved the nutritional status of patients with sarcopenia. Taken together, the mechanism of SJZD in the treatment of sarcopenia may be related to the reduction of peripheral blood inflammation.

On the other hands, the multi-target effect of complex components is the advantage of TCM compatibility in the treatment of diseases, but at the same time, it is also one of the main reasons restricting its development, because useless ingredients cannot play a therapeutic role and will also increase the burden on the liver and kidney.^[32,33]^ Unfortunately, we found that SJZD treatment caused damage to liver function and kidney function. Thus, it is crucial to deeply search for the effective ingredients in SJZD for the treatment of sarcopenia in order to reduce its side effects.

## 5. Conclusion

SJZD can significantly improve the therapeutic efficacy of nutritional therapy and exercise therapy in patients with sarcopenia, and its mechanism is related to the reduction of peripheral blood inflammation in patients with sarcopenia. However, it should be noted that long-term treatment of SJZD also caused liver injury and kidney damage in patients with sarcopenia.

## Author contributions

**Conceptualization:** Yuebing Yue.

**Data curation:** Manfei Xu.

**Funding acquisition:** Yuebing Yue.

**Investigation:** Liyan Qian.

**Validation:** Liping Dou.

**Writing – original draft:** Yuebing Yue, Zheng Xu.

**Writing – review & editing:** Jie Huang.
